# Survey on the management of Pompe disease in routine clinical practice in Spain

**DOI:** 10.1186/s13023-022-02574-5

**Published:** 2022-12-05

**Authors:** Cristina Domínguez-González, Carmina Díaz-Marín, Raúl Juntas-Morales, Andrés Nascimiento-Osorio, Alberto Rivera-Gallego, Jordi Díaz-Manera

**Affiliations:** 1 Neuromuscular Unit, Neurology Department, Hospital Universitario 12 de Octubre, imas12 Research Institute, Biomedical Network Research Center on Rare Diseases (CIBERER), Instituto de Salud Carlos III, Av. de Córdoba, s/n, 28041 Madrid, Spain; 2 Neurology Department, Hospital General Universitario de Alicante Doctor Balmis, Instituto de Investigación Biosanitaria de Alicante (ISABIAL), Alicante, Spain; 3 Neuromuscular Unit, Neurology Department, Hospital Universitario Vall d’Hebron. Peripheral Nervous System Group, Vall d’Hebron Institute of Research (VHIR), Barcelona, Spain; 4 Neuromuscular Unit, Neurology Department, Hospital Sant Joan de Déu, Applied Research in Neuromuscular Diseases, Institut de Recerca Sant Joan de Déu, Center for Biomedical Research Network On Rare Diseases (CIBERER), ISCIII, Barcelona, Spain; 5 Systemic Rare Diseases Unit, Department of Internal Medicine, Hospital Universitario Alvaro Cunqueiro, Vigo, Spain; 6 John Walton Muscular Dystrophy Research Center, Newcastle University Translational and Clinical Research Institute, Newcastle Upon Tyne, UK; 7 Institut de Recerca de l’Hospital de la Santa Creu i Sant Pau, Barcelona, Spain

**Keywords:** Antibodies, Diagnosis, Follow-up, Guidelines, Pompe disease, Treatment

## Abstract

**Background:**

Despite the availability of several clinical guidelines, not all health professionals use their recommendations to manage patients with Pompe disease, a rare genetic disorder involving high-impact therapy. Through several discussion meetings and a survey, the present study aimed to learn about the management of Pompe disease in routine clinical practice in Spain, to improve clinical care in a real-life situation.

**Results:**

The survey was sent to 42 healthcare professionals who manage patients with Pompe disease in their clinical practice. Although most respondents followed the clinical guidelines, clinical practice differed from the expert recommendations in many cases. Approximately 7% did not request a genetic study to confirm the diagnosis before starting treatment, and 21% considered that only two dried blood spot determinations suffice to establish the diagnosis. About 76% requested anti-GAA antibodies when there is a suspicion of lack of treatment efficacy, though a significant percentage of respondents have never requested such antibodies. According to 31% of the respondents, significant impairment of motor function and/or respiratory insufficiency is a requirement for authorizing medication at their hospital. Up to 26% waited for improvements over the clinical follow-up to maintain treatment and withdrew it in the absence of improvement since they did not consider disease stabilization to be a satisfactory outcome.

**Conclusions:**

The results highlight the lack of experience and/or knowledge of some professionals caring for patients with Pompe disease. It is necessary to develop and disseminate simple guidelines that help to apply the expert recommendations better or centralize patient follow-up in highly specialized centers.

## Background

Pompe disease (PD) is an autosomal recessive multisystemic disorder characterized by acid α-glucosidase (GAA) deficiency that leads to lysosomal glycogen storage. The PD phenotype encompasses a continuous clinical spectrum with variable age of onset, progression rate, and severity. The classification of PD differs among authors. According to the age of onset, we can classify it into three groups: 1) Classic infantile-onset PD (IOPD), which includes patients with GAA activity close to 0%, the onset of symptoms during the first year of life, and hypertrophic cardiomyopathy; 2) Childhood or juvenile-onset PD (JOPD), that includes patients diagnosed before age 18; and 3) Adult-onset or late-onset PD (LOPD), that can present after early to late adulthood. Patients in the last two groups usually have residual GAA activity (1–40%) and do not have cardiomyopathy [1–4]. Respiratory failure is the leading cause of morbidity and mortality in JOPD and LOPD. One-third of all patients require ventilatory support before losing the ability to walk independently [3, 5–8].

In 2006, the treatment of PD with recombinant human alglucosidase alfa enzyme (rhGAA) was authorized in Europe. The efficacy and safety of this treatment have been demonstrated in many clinical studies, affording significant improvement of motor function, at least stabilization of lung function, and a prolongation of survival [9–19]. It should be emphasized that patients with less muscle damage at the start of treatment may obtain a more significant benefit, hence the importance of starting treatment as soon as possible [20, 21]. Several clinical studies suggest that starting enzyme replacement therapy (ERT) in a better functional situation indicates a good prognosis; however, the possible benefit of treating presymptomatic patients has not been demonstrated [11, 15, 22, 23]. Considering that almost one-third of all PD patients do not respond to treatment, it is essential to monitor the disease properly, since treatment withdrawal should be considered if no response is observed [24].

Numerous clinical guidelines have been published offering recommendations for diagnosing, treating, and following up on patients with PD [24–28]. For this work, we have taken as a reference the recommendations of the European group of experts on Pompe disease, as it is the most complete and up-to-date guide [24].

It is important to highlight that rhGAA is a high-cost treatment that can be prescribed by any physician in any health center in Spain. Furthermore, the clinical management of PD in Spain is not centralized in reference centers as in other European countries and with other therapies in Spain. For all these reasons, we have conducted a study to know the diagnosis, treatment with rhGAA, and follow-up of patients with PD in real clinical practice in Spain through a 5-question survey. The information obtained will make it possible to determine whether the treatment and follow-up of patients with PD in Spain align with international recommendations, identify inequalities in access to high-impact medications, and identify areas for improvement to optimize their clinical management in the country.

## Results

**Fig. 1:**
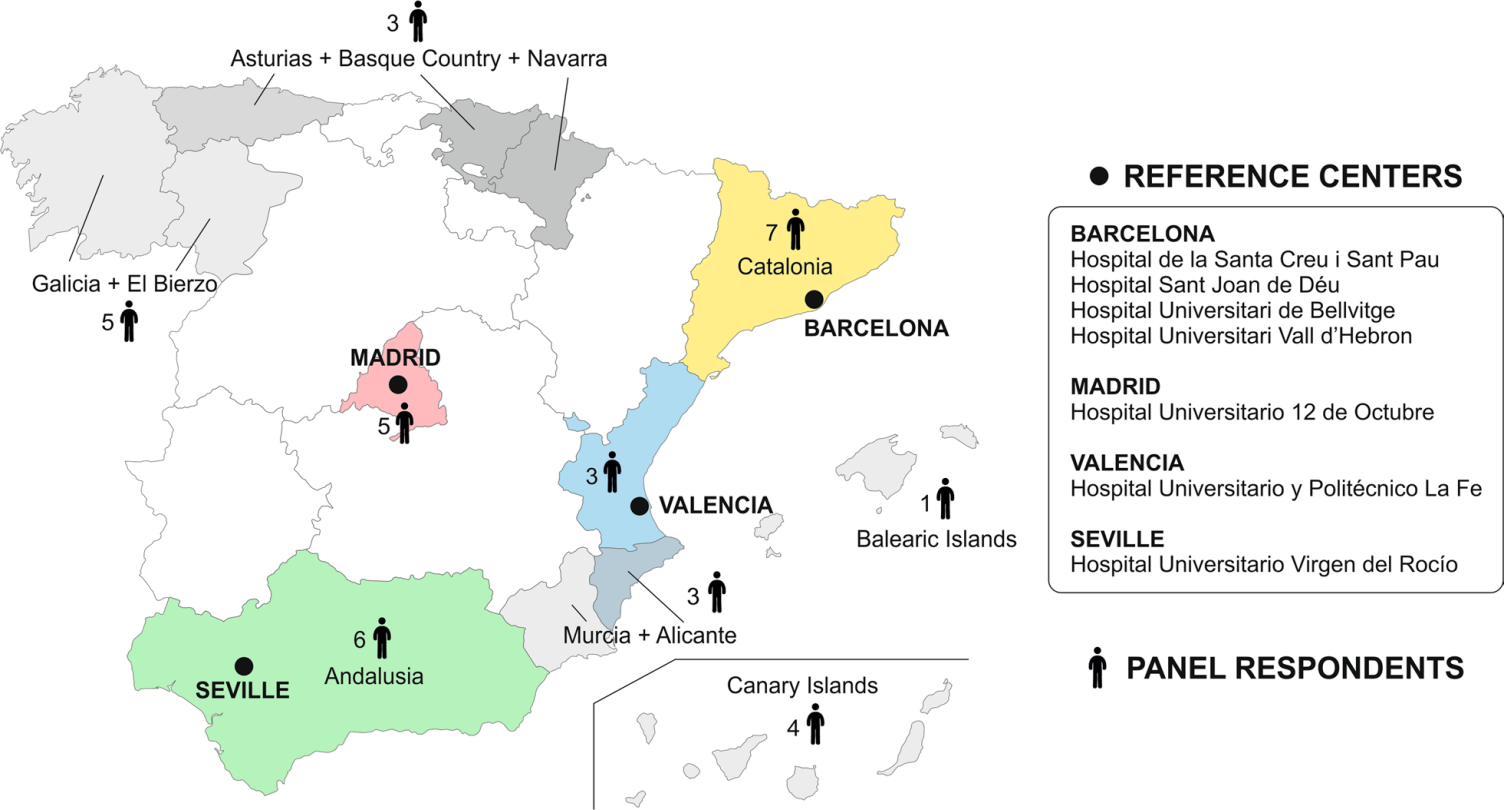
Spanish reference centers across regions in Spain and the origin of the panel respondents that treated patients with adult-onset Pompe disease

The survey was sent to 42 healthcare professionals: 5 treated patients with JOPD (5 pediatricians), and the remaining 37 (8 internal medicine physicians and 29 neurologists) treated patients with adult-onset PD (55% attended more than one). The latter were geographically distributed as follows: 3 in Alicante and Murcia, 6 in Andalusia, 1 in the Balearic Islands, 4 in the Canary Islands, 7 in Catalonia, 5 in Galicia and El Bierzo, 5 in Madrid, 3 in Asturias, the Basque Country, and Navarra, and 3 in Valencia (Fig. 1). The remaining 5 treat JOPD at the National level.

### When do you consider a definitive diagnosis of PD?

Most respondents (97.6%) considered one pathological confirmation in dried blood spot (DBS) testing and the identification of biallelic pathogenic mutations in the genetic study as a confirmatory test for PD (Table 1). Opinions were widely divergent as to whether the presence of reduced enzyme activity in two tissues allows for a diagnosis of PD despite a negative genetic result. Three participants in the survey (7.1%) did not request a genetic analysis to confirm the diagnosis before initiating treatment, and 9 participants (21.4%) indicated that only two DBS determinations were sufficient to diagnose PD.

**Table 1:** Questions proposed in the survey

	N = 42 N (%)
**When do you consider a definitive diagnosis of Pompe disease?**	
When two DBS determinations show pathological values of enzyme activity	9 (21.4)
When a determination of enzyme activity in DBS is pathological and in addition the muscle biopsy shows PAS + vacuoles	17 (40.5)
When DBS is pathological and biallelic pathogenic mutations have been identified in the genetic study	41 (97.6)
When the clinical phenotype is compatible and reduced lymphocyte activity has been demonstrated, even though only one pathogenic mutation has been identified in the genetic study	35 (83.3)
When reduced enzyme activity has been demonstrated in two tissues, even though the genetic study is negative	21 (50.0)
When Pompe disease is suspected, I always request a confirmatory genetic study	39 (92.9)
**Regarding anti-GAA antibodies:**	
I have never requested the determination of anti-GAA antibodies	13 (31.0)
After the initiation of treatment, I request anti-GAA antibody determination on a regular basis	18 (42.9)
If antibodies remain at high titers during follow-up, I consider discontinuing treatment	1 (2.4)
I request antibodies when there is a suspicion of lack of efficacy of the treatment (objective clinical worsening)	32 (76.2)
**What tests do you perform during clinical follow-up to evaluate the response to treatment?**	
MRC scale	39 (92.9)
6MWT	42 (100)
Other timed tests	24 (57.1)
Fatigue scale	19 (45.2)
Activity or quality of life scales	30 (71.4)
Muscle MRI scan	22 (52.4)
Pulmonary function tests	42 (100)
**Do you have to do follow-up reports to keep the medication authorized?**	
No	23 (54.8)
Yes, only occasionally	5 (11.9)
Yes, every 6 months	8 (19.0)
Yes, annually	10 (23.8)
The existence of significant impairment of motor function and/or the presence of respiratory failure is a requirement for the authorization of medication in my hospital	13 (31.0)
Improvement of follow-up parameters is a prerequisite for the maintenance of treatment	11 (26.2)
**In what situations would you consider interrupting or stopping treatment?**	
Never	2 (4.8)
If there are no objective data of stabilization or improvement of motor and/or respiratory function during follow-up	8 (19.0)
If there is evidence of progressive clinical worsening	32 (76.2)

*6MWT* six-minute walk test; *DBS* dried blood spots; *GAA* α-glucosidase, acid; *MRC* Medical Research Council; *MRI* magnetic resonance imaging; *PAS* periodic acid-Schiff

### Regarding anti-GAA antibodies

31% of the respondents had never requested antibodies against GAA, and 42.9% asked for anti-GAA antibody determination regularly after the initiation of treatment. A total of 76.2% requested antibodies when there was a suspicion of a lack of therapy efficacy (objective clinical worsening) (Table 1). Regardless of when these antibodies are requested, almost all the respondents agreed that high antibody titers do not mean treatment should be discontinued (only 2.4% considered high antibody titers as a criterion for discontinuing treatment).

### What tests do you perform during clinical follow-up to assess treatment response?

Most of the respondents chose the Medical Research Council (MRC) scale (92.9%), the six-minute walk test (6MWT) (100%), and pulmonary function tests (100%) (Table 1). The least common tests were activity or quality of life scales (71.4%), other timed tests (57.1%), muscle MRI (52.4%), and fatigue scales (45.2%). Among those using the MRC scale, 64.1% evaluated 10–20 muscles, while 35.9% only explored 5–10 muscles.

### Do you have to do follow-up reports to keep the medication authorized?

54.8% of the respondents answered no, and 54.7% answered yes (23.8% report annually, 19% every six months, and 11.9% only occasionally) (Table 1). The results by region were highly diverse. In Catalonia, all respondents made follow-up reports, versus only one in Alicante and Murcia or Basque Country and Navarra, and none in the Balearic Islands.

According to 31% of the respondents, significant impairment of motor function and/or respiratory insufficiency is a requirement for authorizing medication at their hospital. This affirmation was particularly frequent in Andalusia (50%), Catalonia (57.1%), and in Asturias, Navarre, and the Basque Country (100%). On the other hand, 26.2% considered the improvement of follow-up parameters a prerequisite for maintaining treatment. This statement was particularly frequent in the Canary Islands (75%).

### In what situations would you consider interrupting or stopping treatment?

Most respondents (76.2%) interrupted or stopped treatment when there was evidence of progressive clinical worsening. Only two respondents chose never to interrupt treatment and 19% did so when there was no objective evidence of stabilization or improvement of motor and/or respiratory function during follow-up (Table 1).

## Discussion

The present survey on the management of patients with PD in routine clinical practice yielded highly variable results, with occasional deviation from the recommendations established in the clinical guidelines. The data obtained also reveal the inequality in access to treatment throughout Spain, the absence of homogeneous criteria in accordance with the experts’ recommendations, and the lack of optimization of a treatment with a high economic cost. All of this was noted despite the existence of a robust network of reference centers for rare neuromuscular diseases, which in this case, fail to centralize the management of these low-prevalence, high-complexity patients.

Based on the survey results, several discrepancies have been identified between the usual practice of physicians who care for patients with PD in Spain and the latest published expert recommendations [24–28].

According to the European guidelines, the diagnosis of the disease must be made by a certified laboratory and should be confirmed through enzyme analysis in leukocytes, fibroblasts, or skeletal muscle and/or genetically via mutation analysis (preferably by both methods) [24]. Moreover, although DBS has recently become available and is a good PD screening test, it always requires diagnostic confirmation [29]. However, according to the survey results, some clinicians do not use the criterion of reduced enzyme activity in two tissues to establish the diagnosis of PD in patients with inconclusive genetic testing. Others do not confirm the DBS results with genetic analysis, which can influence the accuracy of diagnosis.

The European consensus recommends discontinuing treatment if high antibody titers are detected that significantly counteract the effect of ERT, which, although rare, is also possible in adults [24]. For this reason, most guidelines recommend determining these antibodies in patients receiving ERT every three months for two years and then annually [25, 28]. In the survey, although many respondents request anti-GAA antibodies when there is a suspicion of lack of treatment efficacy, a significant percentage of those surveyed have never requested them due to the complexity of their determination in real clinical practice. It is interesting to note that recent publications have described that anti-GAA antibody titers decrease as patients are exposed to treatment, and only a few studies have correlated antibody titers to clinical outcomes on a prospective basis [30–33]. Thus, most PD experts agree that the determination of antibodies over time is not very helpful in LOPD patients, and they will rarely interfere with the response to treatment. This justifies the need to update the current recommendations, published in 2017.

During the follow-up of patients, most respondents chose to use pulmonary function tests, the 6MWT, and the MRC scale to evaluate the response to treatment. This is in accordance with the recommendations of several clinical guidelines [26–28]. Other assessments recommended by these guidelines and less frequently employed in clinical practice include the evaluation of nutritional status, the Rasch-built Pompe-specific Activity (R-PAct) scale, and fatigue scales [26–28]. To promote the use of scales for evaluating treatment response, it is necessary to make them more widely known and available.

The European consensus, based on the available evidence and discussion among experts, recommends starting treatment when the patient has symptoms (i.e. the patient should have skeletal muscle weakness or respiratory muscle involvement as observed using clinical assessments) and not starting treatment in pre-symptomatic patients due to the lack of sufficient evidence to support it. Therefore, any patient with symptoms should start treatment [24]. However, a considerable part of those surveyed consider that there must be a significant deterioration in motor function and/or the presence of respiratory failure as a requirement for rhGAA to be authorized in their hospital or that an improvement in monitored parameters is required to maintain treatment.

To maintain authorization of the treatment, the European guidelines state that both an improvement and stabilization in motor and/or respiratory function suggests that the treatment is beneficial and should be continued [24]. They recommend stopping therapy in cases of serious adverse events that cannot be controlled, severe comorbidity that limits the patient’s life expectancy, or if the patient should decide [26]. In addition, they recommend stopping treatment if the patient shows a substantial deterioration in motor and respiratory functions but restarting it if the progression of the disease accelerates after stopping the ERT [24]. A large majority of respondents in our survey agreed with these recommendations. However, 26.2% of those surveyed in this study do not consider stabilization a beneficial effect of the ERT and recommend its suspension in these cases.

The variety in the responses suggests that there is no standardized diagnosis or care protocol in the country and that published guidelines are not always followed. Consequently, patient care is based on the expertise of each physician. On the other hand, some answers seem to depend more on the center than on the health care region involved and, in many cases, the authorization to start and stop ERT does not rely only on the doctor but also on the pharmacy committee of the center, which may not follow the clinical guideline recommendations.

The discrepancies observed in our study with the published evidence highlight the need to establish clear and straightforward diagnostic and therapeutic guidelines for this disease or to restrict the indication and follow-up of high-impact treatments to reference centers for PD. In Spain, there are seven major reference centers for rare neuromuscular diseases designated by the Spanish Ministry of Health (Fig. 1) [34]. These centers can also be essential in avoiding inequalities in access to medicines depending on the area of residence.

The main limitations of this study are those inherent to studies based on online surveys, i.e., differences in understanding and interpretation of the questions, closed-ended questions, non-response bias, and lack of personalization, among others.

## Conclusions

The present survey results on the management of PD in Spain (a rare disease with a high-impact treatment) reveal some professionals’ lack of experience or knowledge. Developing and disseminating simple guidelines that help understand and follow the recommendations is necessary. It might even be interesting to suggest that the follow-up of these patients be carried out in the existing reference centers. The inequity detected in access to rhGAA and the discrepancies with the current evidence justify the need to involve the Administration in the homogenization and optimization of the clinical management of patients with PD.

## Methods

### Study design

The present study involved a survey to gather opinions on the management of PD in real clinical practice in Spain. The study was conducted in several phases: 1) project definition and creation of the scientific committee; 2) generation of materials to be presented at meetings based on the current state of the art in the diagnosis and treatment of PD; 3) a total of 10 online meetings conducted by an expert to discuss the diagnosis, treatment indication, clinical management, and follow-up of patients with PD and to identify controversies; 4) creation and distribution of an online survey to collect the opinion of the respondents on the management of PD, and 5) a final meeting with the scientific committee to discuss the results.

### Participants

The study involved three types of professionals: a scientific committee, a technical team, and a panel of respondents. The scientific committee consisted of 10 experts in the management of PD, whose role was to lead several discussion meetings and to develop a survey to know the usual clinical practice in the management of PD. The technical team, which directed and supervised the entire process, was responsible for the instrumental implementation of the method (literature search, survey distribution, analysis of the responses, and statistical interpretation of the survey). The scientific committee chose the panel of respondents among health professionals treating patients with PD. This panel consisted of 42 professionals (8 internal medicine physicians, 29 neurologists, and five pediatricians) with experience in the management of PD from all over Spain (Fig. 1).

### Discussion meetings

Through ten meetings with healthcare professionals who attend to patients with PD, the scientific committee presented the most recent scientific evidence about this disease, showing the main recommendations of clinical guidelines on the diagnosis, treatment, and follow-up of patients. During these meetings, the management of the disorder was discussed, and the most relevant controversies were identified and collected in a worksheet. Each session was held online, lasted 2 h, was moderated by a scientific committee member, and was attended by 4–6 physicians.

### Survey

Based on the controversies identified in the discussion meetings, a 5-question survey of diagnostic and treatment practices in patients with PD was developed. Each question included several answers, and the respondents could choose any. The purpose of the survey was to ascertain the disparity of opinions about the management of the disease and to identify opportunities for improvement in the diagnosis, treatment, and follow-up of patients with PD. The results were analyzed anonymously, without differentiating between the professionals’ responses belonging to reference centers and the rest.

## Data Availability

Not applicable.

## Abbreviations

6MWT: Six-minute walk test
DBS: Dried blood spot
ERT: Enzyme replacement therapy
GAA: Acid α-glucosidase
IOPD: Infantile-onset PD
JOPD: Juvenile-onset PD
LOPD: Late-onset PD
MRC: Medical Research Council
MRI: Magnetic resonance imaging
PD: Pompe disease
R-PAct: Rasch-built Pompe-specific Activity
rhGAA: Recombinant human alglucosidase alfa enzyme
